# The Factor Structure and External Validity of the COPE 60 Inventory in Slovak Translation

**DOI:** 10.3389/fpsyg.2021.800166

**Published:** 2022-02-28

**Authors:** Júlia Halamová, Martin Kanovský, Katarina Krizova, Katarína Greškovičová, Bronislava Strnádelová, Martina Baránková

**Affiliations:** ^1^Faculty of Social and Economic Sciences, Institute of Applied Psychology, Comenius University in Bratislava, Bratislava, Slovakia; ^2^Faculty of Social and Economic Sciences, Institute of Social Anthropology, Comenius University in Bratislava, Bratislava, Slovakia

**Keywords:** coping, cope, factor analysis, validity, psychometric analysis

## Abstract

The COPE Inventory (Carver et al., 1989) is the most frequently used measure of coping; yet previous studies examining its factor structure yielded mixed results. The purpose of the current study, therefore, was to validate the factor structure of the COPE Inventory in a representative sample of over 2,000 adults in Slovakia. Our second goal was to evaluate the external validity of the COPE inventory, which has not been done before. Firstly, we performed the exploratory factor analysis (EFA) with half of the sample. Subsequently, we performed the confirmatory factor analysis with the second half of the sample. Both factor analyses with 15 factor solutions showed excellent fit with the data. Additionally, we performed a hierarchical factor analysis with fifteen first-order factors (acceptance, active coping, behavioral disengagement, denial, seeking emotional support, humor, seeking instrumental support, mental disengagement, planning, positive reinterpretation, religion, restraint, substance use, suppression of competing activities, and venting) and three second-order factors (active coping, social emotional coping, and avoidance coping) which showed good fit with the data. Moreover, the COPE Inventory’s external validity was evaluated using consensual qualitative research (CQR) analysis on data collected by in-depth interviews. Categories of coping created using CQR corresponded with all COPE first-order factors. Moreover, we identified two additional first-order factors that were not present in the COPE Inventory: self-care and care for others. Our study shows that the Slovak translation of the COPE Inventory is a reliable, externally valid, and well-structured instrument for measuring coping in the Slovak population.

## Coping Conceptualization

Coping is defined as “efforts to prevent or diminish threat, harm, and loss, or to reduce the distress” that we experience during times of adversity (Carver, 2013a). Well-known methods of measuring coping include the ways of coping questionnaire (Folkman and Lazarus, 1985), coping strategies questionnaire (Rosenstiel and Keefe, 1983), coping inventory for stressful situations (Endler and Parker, 1990), and the COPE Inventory (Carver et al., 1989) which is used most frequently (Kato, 2015). The COPE Inventory (Carver et al., 1989) assesses a variety of functional and dysfunctional coping strategies utilized by individuals in their response to stress.

The COPE Inventory was developed as a theory-based measure in contrast to previous measures of coping that were constructed empirically (cf. Folkman and Lazarus, 1985; McCrae and Costa, 1986). The transactional model of stress and coping by Lazarus and Folkman (1984) and the behavioral self-regulation model by Carver and Scheier (1981) informed the development of the COPE first-order factors. The transactional model (Lazarus and Folkman, 1984) describes the process of coping as being dependent upon the individual’s cognitive appraisal of the stressor, the relevance the individual attributes to the stressor, and the resources available to the individual. Coping, and subsequently the outcome of coping, are influenced by the transactional nature of the individual’s resources and the environmental stressors (Lazarus and Folkman, 1984). The COPE also reflected Carver and Scheier’s (1982) understanding of self-regulatory behaviors that, through feedback control processes, lead to positive and negative feelings and goal-directed action. The theoretical construction of the measure allowed for the identification of 14 conceptually distinct first-order factors that were confirmed by factor analysis and that clearly differentiated specific coping responses (Carver et al., 1989). An additional subscale, humor, typified as making light of the situation, was added later (Deisinger et al., 1996). The responses included in the inventory were ones individuals described using to lower distress and reduce the risk of harm and loss associated with stressful experiences (Carver, 2013a).

## The COPE Inventory

Altogether, the COPE Inventory multidimensional measure consists of 60 items and 15 first-order factors (4 items each). The first-order factors are based on theoretical assumptions about functional coping and previous research findings that indicated fostering and hindering factors of adaptive coping (Carver et al., 1989). The first-order factors are: 1. Acceptance: being accepting of the situation; 2. Active coping: performing specific actions to deal with the situation; 3. Behavioral disengagement: reactive refusal to deal with stress; 4. Denial: denying the reality of the situation; 5. Seeking emotional support: relying on others for empathy and understanding; 6. Humor: joking about the situation; 7. Seeking instrumental support: seeking instrumental help from others, such as advice or information; 8. Mental disengagement/self-distraction: doing activities that distract the person from unpleasant thoughts related to the problem; 9. Planning: strategizing on how to deal with a stressful situation; 10. Positive reinterpretation: finding positives in a stressful situation; 11. Religion: using religious activities to cope, such as praying; 12. Restraint: making sure one does not respond to stress in a reactive way; 13. Substance use: using substances to deal with a distressing situation; 14. Suppression of competing activities: intentionally avoiding activities that do not help the person deal with the problem; and 15. Venting: sharing negative emotions.

On a 4-point Likert scale (1 = I usually do not do this at all; 4 = I usually do this a lot), the respondents are asked to mark the frequency with which they engage in different coping responses. The COPE Inventory is available in three formats; the dispositional format asks about typical coping responses, whereas the two time-limited versions ask about coping that occurred at a specific time or still in use (Carver, 2013b). An abbreviated measure, Brief COPE (Carver, 1997) was developed to address the length of the measure. The abbreviated version includes 28 items and 14 first-order factors (2 items each). Brief COPE was validated for different populations, including a community sample affected by a hurricane (Carver, 1997). Several language versions of Brief COPE exist, for example Spanish (Perczek et al., 2000), Korean (Kim and Seidlitz, 2002), French (Muller and Spitz, 2003), and Chilean Spanish (García et al., 2018).

## Psychometric Properties of the COPE Inventory

The COPE 60 Inventory is reported to have good psychometric properties (Carver et al., 1989). Fifteen first-order factors were confirmed by the factor analysis (e.g., Carver et al., 1989; Deisinger et al., 1996). In the original investigation, internal consistency, α, of the first-order factors in two student samples ranged between 0.62 and 0.92, excluding the mental disengagement scale (α = 0.45). Test-retest reliability coefficients (*r*) ranged from between 0.46 and 0.86 (Carver et al., 1989).

Convergent and discriminant validity was determined by correlating the COPE first-order factors with different personality traits, such as optimism, hardiness, self-esteem, trait anxiety, social desirability, and perceived control (Carver et al., 1989). The associations between the adaptive and less adaptive coping subscale and various personality traits were as expected. For example, active coping was positively correlated with optimism (*r* = 0.32), control (*r* = 0.21), self-esteem (*r* = 0.27), and negatively with anxiety (*r* = − 0.25).

The measure was validated in different languages and across various populations. The COPE Inventory has been translated into Chinese (Hsu, 2003), Spanish (Perczek et al., 2000), Estonian (Kallasmaa and Pulver, 2000), Russian (Garanyan and Ivanov, 2010), Arabic (Alghamdi, 2020), Romanian (Craşovan and Sava, 2013), and French (Desbiens and Fillion, 2007). A recent utilization of the COPE Inventory in a Romanian convenience sample yielded internal consistency ranging between α = 0.72 and α = 0.84 for a four-factor solution (Craşovan and Sava, 2013). Further, acceptable psychometric properties were reported for other translations, for example for Russian (between α = 0.53 and α = 0.90; Garanyan and Ivanov, 2010), Arabic (between α = 0.75 and α = 0.84; Alghamdi, 2020), Estonian (between α = 0.49 and α = 0.95; Kallasmaa and Pulver, 2000), and French (between α = 0.61 and α = 0.91; Desbiens and Fillion, 2007).

A Slovak translation has been utilized to assess coping in relation to adolescent personality dimensions (Fickova, 2009); however, the measure was used without being validated. The internal consistency range in the sample of 200 Slovak adolescents was acceptable, from α = 0.60 to α = 0.94, excluding active coping (α = 0.40). The author did not explain what could have potentially contributed to the low internal consistency of the active coping items in the adolescent sample (Fickova, 2009).

## Factor Structure of the COPE Inventory

Several coping responses in the measure were relevant to problem-focused and emotion-focused coping as delineated by Folkman and Lazarus (1985). Problem-focused coping emphasized action-taking during stress response, whereas emotion-focused coping pertained to emotional distress management (Carver et al., 1989). Specifically, the problem-focused responses in the measure were active coping, planning, suppression of competing activities, restraint coping, and instrumental social support, whereas the emotion-focused responses were positive reinterpretation, acceptance, turning to religion, and emotional social support (Carver et al., 1989). However, factor loadings showed that the coping responses could not be easily divided into problem-focused and emotion-focused aggregates, since some responses loaded on the same second-order factor despite their different coping focus (Carver et al., 1989; Litman, 2006). This was confirmed by several investigations that utilized the measure (Stowell et al., 2001; O’Connor and O’Connor, 2003).

In their original article, Carver et al. (1989) identified four second-order factors: 1. coping focused on the problem which included the following first-order factors: active coping, planning, and suppression of competing activities, 2. emotion-focused coping which included these first-order factors: seeking instrumental social support, seeking emotional social support, and venting, 3. disengagement which included these first-order factors: denial, mental disengagement, and behavioral disengagement, and 4. acceptance which included these first-order factors: acceptance, restraint coping, and positive reinterpretation. Further utilization of the measure, however, revealed inconsistencies in the higher-order factor structure. Although some studies confirmed a four second-order factor structure, albeit with slight differences between factor loadings on problem-based and emotion-based coping (Fontaine et al., 1993; O’Connor and O’Connor, 2003; Craşovan and Sava, 2013), other studies identified only three factor loadings (Stowell et al., 2001; Litman, 2006), and yet other studies reported five-factor loadings (Sica et al., 1997). Thus, it is believed that the specific coping responses are not mutually exclusive, as individuals might use a variety of adaptive and less adaptive coping responses in conjunction (Carver et al., 1989). Based on previous investigations, Litman (2006) suggested that it might be more meaningful to differentiate between socially supported and self-sufficient coping styles, although the author of the measure recommends examinations of each subscale separately (Carver, 2013b).

## The Aim of the Current Study

The psychometric properties of the theoretically constructed COPE 60 Inventory have been tested in numerous samples across different settings. Several translations of the COPE Inventory have not yielded satisfactory psychometric properties; instead, many previous studies reported low internal consistency for at least one subscale (Kallasmaa and Pulver, 2000; Garanyan and Ivanov, 2010), including a previous study conducted in a Slovak sample (Fickova, 2009). Similarly, previous studies identified different factor structures, identifying anywhere between three to five factors (Sica et al., 1997; O’Connor and O’Connor, 2003; Litman, 2006). Study 1 aims to determine the psychometric properties and factor structure of a Slovak translation of the COPE Inventory in a representative sample of Slovak adults. Furthermore, to the best of our knowledge, the COPE Inventory has not been externally validated; therefore, our second goal is to conduct a qualitative validity check to see whether our identified factors are sufficient and exhaustive. External validity explains how well the findings of a questionnaire apply to other settings (Patino and Ferreira, 2018). Our aim, therefore, was to see whether the coping strategies in the COPE Inventory sufficiently and exhaustively represented the coping strategies utilized by participants during a stressful situation. The COPE Inventory is a theory-driven instrument and no previous research has tested its validity using a qualitative analysis. Mixed method analysis using information obtained from multiple sources, *via* self-reported questionnaires and semistructured interviews and the like has become more and more common (Clark and Watson, 2019). Qualitative data are “analyzed by identifying the fitness between the observed pattern and the expected pattern, to test the theory’s validity” (Vargas-Bianchi, 2020, p. 3), which makes qualitative analysis uniquely suitable for a validity investigation of a theory-driven questionnaire. Similarly, “theories are brought into and produced by qualitative research projects and they link that project to wider bodies of knowledge [at the conceptual or even theoretical level we can talk about analytic or conceptual generalizability and the possibility of transferring our ‘findings’ to other settings or situations]” (Giacomini, 2010, p. 148). It is important to test the external validity of a theory-driven questionnaire applied in an entirely different context to ensure generalizability across situations (the extent to which we can generalize from the situation constructed in the questionnaire to real-life situations (see Aronson et al., 2007). Therefore, Study 2 seeks to determine whether the 15 first-order factors of the COPE Inventory are represented in participants’ narratives of coping.

## Study 1

### Representative Sample

A sample representative of the Slovak population in terms of age, gender, region, and population density was collected a data collection company, utilizing stratified sampling. Our sample consisted of 2,077 participants, out of which 1,114 participants were women and 963 were men). The mean age was 47.16 years (*SD* = 17.06) and the ages ranged from 18 to 89 years. All the participants were Slovak citizens. To participate in our research, participants had to complete an online informed consent form. Data were collected in accordance with the ethical standards of the institutional and/or national research committee and in accordance with the 1964 Helsinki Declaration and its later amendments or comparable ethical standards. The study’s protocol was approved by the Ethical Committee of the Faculty of Social and Economic Sciences at Comenius University, Bratislava.

### Measures

#### The COPE Inventory

The COPE Inventory was developed by Carver et al. (1989) based on theoretical assumptions about functional coping. The COPE consists of 60 items that are divided into 15 first-order factors with 4 items in each.

The first-order factors are 1. Acceptance (e.g., “I learn to live with it.”), 2. Active coping (e.g., “I do what has to be done, one step at a time.”), 3. Behavioral disengagement (e.g., “I just give up trying to reach my goal.”), 4. Denial (e.g., “I pretend that it hasn’t really happened.”), 5. Seeking emotional support (e.g., “I discuss my feelings with someone.”), 6. Seeking instrumental support (e.g., “I talk to someone who could do something concrete about the problem.”), 7. Mental disengagement/self-distraction (e.g., “I turn to work or other substitute activities to take my mind off things.”), 8. Planning (e.g., “I make a plan of action.”), 9. Positive reinterpretation (e.g., “I try to grow as a person as a result of the experience.”), 10. Religion (e.g., “I put my trust in God.”), 11. Restraint (e.g., “I hold off doing anything about it until the situation permits.”), 12. Substance use (e.g., “I drink alcohol or take drugs, in order to think about it less.”), 13. Suppression of competing activities (e.g., “I put aside other activities in order to concentrate on this.”), 14. Venting (e.g., “I get upset and let my emotions out.”) and 15. Humor (e.g., “I laugh about the situation.”).

The COPE is the most commonly used measure of coping behavior (Kato, 2015; Voronin et al., 2020). It was previously translated and utilized in a study by Fickova (2009); however, Fickova’s (2009) translation was not validated and one of the reliability coefficients was very low (α = 0.40; Fickova, 2009). Therefore, we decided to retranslate the measure; the authors of this study served as the expert panel. The final version of the Slovak translation of the COPE Inventory can be found in Supplementary Appendix 4.

### Data Analysis

To analyze the data, we used confirmatory factor analysis (CFA) with the weighted least squares mean and variance adjusted method (WLSMV) as an estimator and target rotation specifying the theory-driven loadings while permitting for small crossloadings. WLSMV estimator is recommended for use with ordinal items (Beauducel and Herzberg, 2006; Bandalos, 2014). We used Mplus version 8.4 for statistical analysis (Muthén and Muthén, 2017).

## Results

### Factor Analysis of the COPE Inventory

#### Exploratory and Confirmatory Factor Analyses With WLSMV

Since our representative sample consisted of about 2,000 people, we split it in half to perform both the exploratory (EFA) and confirmatory factor analyses (CFA). Asparouhov and Muthén (2009) stressed the risks of performing CFA when the factor structure is not known or uncertain, and that in these cases it is best to perform both exploratory and confirmatory analyses. Additionally, the factorial structure of an instrument validated in different settings and languages cannot be relied upon in a Slovak setting.

Results of EFA showed excellent fit of the model with data as follows χ^2^(975) = 59458.945, *p* < 0.001, CFI = 0.993, TLI = 0.987, SRMR = 0.055, and RMSEA = 0.048, 90% CI [0.047, 0.049], and average factor loadings (*M* = 0.484, see Supplementary Appendix 1) ranging from 0.108 to 0.939. The fifteen-factor CFA model also had an excellent fit with the data: χ^2^(1,605) = 5,355.368, *p* < 0.001, CFI = 0.938, TLI = 0.932, SRMR = 0.055, and RMSEA = 0.047, 90% CI [0.046, 0.049]. Factor loadings are in Supplementary Appendix 2. Both the fit indices and factor loadings supported the fifteen-dimensional model of the COPE inventory (see Supplementary Appendix 2).

#### Exploratory Factor Analysis With the COPE Inventory’s Scores

In accordance with the analytical procedures utilized in the study by Litman (2006), we evaluated the COPE Inventory scores using iterated principal axis factor analysis with promax rotation allied with the squared multiple correlation for the communality estimate. The factor extraction yielded three factors: Factor 1 representing active coping, Factor 2 representing social emotional coping, and Factor 3 representing avoidant coping (Table 1). The three factors together explained 54.97% of variance (Factor 1 = 37.79%, Factor 2 = 12.26%, and Factor 3 = 4.92%). The Kaiser–Meyer–Olkin (KMO) test was 0.902, indicating that the data was well-suited for factor analysis. Further, Bartlett’s test of sphericity was significant at *p* < 0.0001, *X*^2^ (105) = 16,158.62 (see Table 1). We performed the EFA with the total score of each of the first-order factors to compare the results with the previous studies (e.g., Stowell et al., 2001; Litman, 2006), even though this procedure is not parsimonious and does not adhere to current methodological practice. Therefore, we also ran a second-order CFA in which we tested simultaneously both the first level (15 first-order factors) and second level (3 second-order factors) structure (Table 2). The second-order CFA model had good fit with the data: χ^2^(1692) = 7,922.429, *p* < 0.001, CFI = 0.898, TLI = 0.893, SRMR = 0.079, and RMSEA = 0.060, 90% CI [0.058, 0.061]. Factor loadings are in Table 2. Both the CFA factor analyses supported the three second-order factor models of the COPE Inventory with the same first-order factors loading in the same second-order factors.

**TABLE 1:** Exploratory factor analysis (EFA) factor loadings of three-factor model of the COPE Inventory scores.

The COPE Inventory first-order factors	Factor 1	Factor 2	Factor 3
Positive reinterpretation	**0.868**	–0.104	–0.028
Active coping	**0.876**	0.055	–0.123
Restraint	**0.800**	0.011	0.074
Planning	**0.777**	0.129	–0.096
Acceptance	**0.681**	0.028	0.063
Suppression of competing activities	**0.513**	0.200	0.199
Seeking emotional support	0.048	**0.867**	–0.110
Seeking instrumental support	0.217	**0.738**	–0.144
Venting	0.043	**0.564**	0.228
Religion	–0.023	**0.370**	0.071
Denial	–0.027	0.013	**0.776**
Behavioral disengagement	–0.200	0.289	**0.658**
Substance use	–0.148	0.001	**0.592**
Humor	0.282	–0.316	**0.515**
Mental disengagement/Self-distraction	0.282	0.074	**0.499**

*Factor 1 = active coping; Factor 2 = social-emotional coping; Factor 3 = avoidance coping. Bold items belonging to the particular factor.*

**TABLE 2:** Factor loadings of the second-order CFA simultaneously testing both the first level (15 first-order factors) and second level (3 second-order factors) structure of the COPE.

	Active coping	Social-emotional coping	Avoidance coping
Positive reinterpretation and growth	0.856		
Active coping	0.970		
Restraint	0.978		
Acceptance	0.823		
Suppression of competing activities	0.948		
Planning	0.943		
Focus on and venting of emotions		0.817	
Use of instrumental social support		0.966	
Religious coping		0.413	
Use of emotional social support		0.905	
Mental disengagement			0.999
Denial			0.781
Humor			0.439
Behavioral disengagement			0.798
Substance use			0.427

### Reliability

Coefficients of reliability (Cronbach’s alpha) calculated for the first-order factors are presented in Table 3. They ranged from 0.55 to 0.95 with good values except for Mental Disengagement. We also calculated the McDonald’s omega (the composite reliability) for the second-order factors—the Omega total (all explained variance) and the Omega hierarchical (variance explained by a strong single general factor, see Rodriguez et al., 2016): Active coping: Cronbach’s alpha = 0.93, Omega Total = 0.95, Omega Hierarchical = 0.78, Social Emotional coping: Cronbach’s alpha = 0.90, Omega Total = 0.94, Omega Hierarchical = 0.70, Avoidance coping: Cronbach’s alpha = 0.88, Omega Total = 0.92, Omega Hierarchical = 0.71.

**TABLE 3:** Reliability of the Slovak COPE Inventory.

COPE first-order factors	1	2	3	4	5	6	7	8	9	10	11	12	13	14	15
Cronbach’s alpha	0.76	0.79	0.74	0.70	0.86	0.87	0.82	0.55	0.78	0.78	0.95	0.70	0.91	0.69	0.77

*1 = Acceptance; 2 = Active coping; 3 = Behavioral disengagement; 4 = Denial; 5 = Seeking emotional support; 6 = Humor; 7 = Seeking instrumental support; 8 = Mental disengagement; 9 = Planning; 10 = Positive reinterpretation; 11 = Religion; 12 = Restraint; 13 = Substance use; 14 = Suppression of competing activities; 15 = Venting.*

## Study 2

### Non-representative Sample

First, a convenience sample was collected by sharing a recruitment post on social media. The convenience sample consisted of 1,683 participants (1,129 women and 543 men, 11 chose not to disclose) with mean age 31.02 years (*SD* = 11.99) and age range between 18 and 77 years. All respondents were Slovak citizens and completed an online informed consent form before participating.

Second, we selected six participants from the convenience sample who had the highest scores in coping skills in the majority of the COPE first-order factors. We chose these six participants based on the expectation that people with highly adaptive coping skills engage in a variety of coping strategies and utilize different coping mechanisms to deal with distress. The maximum score for each COPE subscale was 14 points, and we used 10 points as the cut-off score. Our first selected participant had a score higher than 10 points in six first-order factors, our second and third participants had scores higher than 10 in five first-order factors, and our fourth, fifth, and sixth participants had a score higher than 10 in four first-order factors. One participant was selected to check our data for saturation after the qualitative analysis was finalized. This participant was selected randomly from a pool of eight participants who had a score higher than 10 points in three first-order factors.

The participants were: a 20-year-old female university student, a 23-year-old female had completed secondary education, a 24-year-old female university student, a 27-year-old female who had completed university education, a 36-year-old female who had completed university education, a 38-year- old woman who had completed university education, a 46-year-old female who had completed university education.

Since all these highly performing participants were women, we decided to balance the sample by adding two male participants who had the most variability in high coping skills determined by scoring higher than 10 in two first-order factors of the COPE Inventory. The participants were: a 21-year-old male university student and a 29-year-old male who had completed university education. These two male participants also served as a check on our data for saturation. Altogether, the sample for Study 2 included nine participants.

Data were collected in accordance with the ethical standards of the institutional and/or national research committee and in accordance with the 1964 Helsinki Declaration and its later amendments or comparable ethical standards. The study’s protocol was approved by the Ethical Committee of the Faculty of Social and Economic Sciences at Comenius University, Bratislava.

### Data Collection

Data for Study 2 were collected by conducting two in-depth interviews with each participant focused on the participant’s coping during the COVID-19 pandemic. Both interviews together lasted approximately 3 hours. The interviews were semistructured with open-ended questions. The interview protocol consisted of the following areas: the participant’s prepandemic functioning, the participant’s pandemic functioning, stressful situations and the ways the participant coped with these stressful situations, and the participant’s evaluation of the pandemic’s effect on their life.

### Data Analysis

Nine in-depth interviews were transcribed and analyzed using consensual qualitative analysis (CQR; Hill et al., 1997). The five-member research team consisted of four post-doc researchers and one full professor as auditor. Members of the research team analyzed the data by first creating categories, then subdomains, and then domains. Next, members of the research team discussed the domains they created given to the auditor for feedback. The final categorizations were obtained by a consensus among the researchers (Hill et al., 1997).

## Results

### The External Validity of the COPE Inventory

**FIGURE 1:**
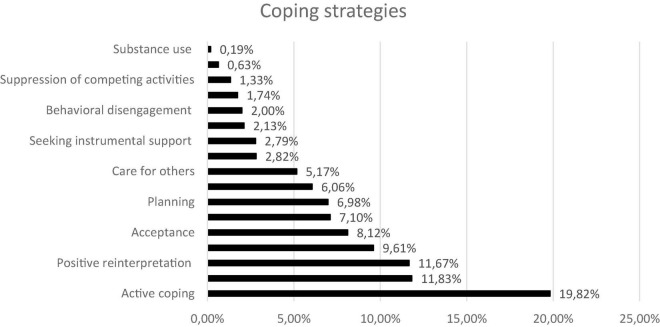
Frequency of the various coping strategies based on the COPE.

The coping created using CQR corresponded to all the COPE first-order factors. Each of the first-order factors was present in the participants’ interviews; albeit with differing frequencies. See Figure 1 for the frequency of all categories. Moreover, we identified two additional categories one of these consisted of first-order factors that had not been included in the COPE Inventory, and which we named 16. Self-care, which is related to managing stress level by engaging in pleasant and unpleasant activities and own fulfilling needs; and 17. Care for others, which is linked to helping others to relieve their stress in order to relieve their own stress. Illustrative examples of the various first-order factor categorizations are given in Supplementary Appendix 3. More example of the new categories can be found in Supplementary Appendix 5.

## Discussion

The goal of this research study was to analyze the factor structure of the COPE Inventory (Carver et al., 1989) and to test the external validity of the scale, which had not been done before.

In our representative sample ranged from 0.55 to 0.95. Only the mental disengagement subscale had a lower than acceptable reliability coefficient. Our results echo the findings of the original English study by Carver et al. (1989), who calculated internal consistency for mental disengagement subscale to be 0.45, and also the findings from a Russian sample (Garanyan and Ivanov, 2010) and from a Romanian sample (Craşovan and Sava, 2013) that determined the Cronbach’s Alpha’s value for mental disengagement to be 0.54.

Similar to our reliability findings, the lowest factor loadings were found for several items of the mental disengagement subscale. The factor loadings ranged from 0.450 to 0.519 for three items (no. 16, 31, 43). By contrast, the factor loading for item 2 was 0.683, suggesting that this item was well-formulated. The second lowest value was identified in the denial subscale a loading of 0.573. However, these loadings are still well above the generally accepted level (0.30).

Based on our results, we recommend reformulating items 16, 31, and 43 of the mental disengagement subscale since they appear to differ in how specific they are about possible disengaging activities. For example, item number 31 states: “I go to movies or watch TV, to think about it less.” Based on our results, we believe that this statement could be potentially misleading, since participants might misinterpret the purpose of engaging in the activity described in the statement and think about watching tv as a relaxation technique rather than as a disengagement activity. It appears that the well-formulated items (e.g., item no. 2) are the more general ones that do not mention specific activities that people use for disengagement.

Both the EFA and CFA factor analyses of the COPE Inventory showed a good fit with the data and high factor loadings for all fifteen first-order factors. Our results are consistent with previous research (e.g., Carver et al., 1989; Deisinger et al., 1996). However, we did not confirm that the COPE Inventory had a two second-order factor structure dividing coping strategies into problem-focused and emotion-focused strategies, as proposed by Folkman and Lazarus (1985). Our factor analysis of the subscales showed three second-order factors, which is in line with Stowell et al. (2001) and Litman (2006). Also the higher-order factor analysis showed good fit for the three second-order factors. In our study, the first identified second-order factor labeled “Active” consisted of positive reinterpretation, active coping, restraint, acceptance, suppression of competing activities and planning; the second identified second-order factor labeled “Social Emotional” comprised venting, seeking instrumental support, religion, and seeking emotional support; and the third identified second-order factor labeled “Avoidance” (Stowell et al., 2001) consisted of mental disengagement, denial, humor, behavioral disengagement, and substance use. Stowell et al. (2001) and Litman (2006) reported almost identical results in terms of the factor structure and first-order factors identified in each factor.

In Litman’s (2006) study, two COPE factors reflected adaptive coping, namely self-sufficient coping and socially supported coping and one factor reflected non-adaptive coping, namely avoidant coping. This corresponded with our results: self-sufficient coping could be found in our “Active” factor. We also agree with Litman (2006) that problem-focused and emotion-focused strategies can be observed across all factors and are not factor-specific. Furthermore, it seems more relevant to consider three factors rather than two factors because both problem-focused and emotion-focused coping are believed to have positive benefits on health (e.g., McQueeney et al., 1997; Schoenmakers et al., 2012) in comparison to avoidance. Thus, the question of adaptive coping may not depend on whether problem-focused or emotion-focused coping styles are preferred so much as on whether active and social-emotional strategies are used instead of avoidant strategies (Stowell et al., 2001).

Two first-order factors are loaded differently in our study compared to Stowell et al. (2001) and Litman (2006): religion and humor. In our sample, religious coping was linked to the Social emotional factor, a finding that appears to be specific to the Slovak population. According to Bunčák (2002, p. 152), Slovakia is among the “countries which maintained the high level and the long-term continuity of the positive relation to religious faith.” This might explain why in Slovakia “religion” as a means of coping belonged to the Social Emotional factor unlike in Stowell et al. (2001) who identified religion as part of the active factor and to Litman (2006) in which religion did not reach a satisfactory loading. Similarly, Carver et al. (1989) agreed that people may turn to religion for many different reasons. Therefore, it is difficult to know whether religion is used as an adaptive or non-adaptive coping strategy (Stowell et al., 2001).

Humor was another subscale that loaded in a different factor in our study from in Stowell et al.’s (2001) and Litman’s (2006) investigations. This could be attributable to cultural differences in utilizing humor as a coping skill, since in Slovakia, negative and self-deprecating humor is common. Overall, Study 1 shows that the COPE inventory is a reliable measure whose original factor structure (Carver et al., 1989) is supported in the Slovak population. Since the sample in Study 1 was representative, we can interpret our results as being generalizable to the Slovak population.

In Study 2, the consensual qualitative analysis showed that all the first-order factors of the COPE Inventory were supported in our data. In our non-representative sample consisting of participants with the highest coping scores across the majority of first-order factors, the most frequently used coping strategies were active coping, which represented nearly 20% of all coping strategies, and positive reinterpretation and seeking emotional support, each of which represented nearly 12%. Our newly created subscale labeled “self-care” was represented in nearly 9% of the data; its high frequency further supports the need to add this subscale to the new revision of the COPE Inventory. Our results showed that our participants were more likely to use adaptive coping strategies, such as active coping, than non-adaptive strategies, such as substance use.

Interestingly, the two new first-order factors that we identified in our data, namely self-care and care for others, included our participants’ descriptions of using their mentalization skills to recognize internal distress related to their own feelings of being unwell or suffering, or the internal distress experienced when their loved one is suffering. Our participants described the ways in which they dealt with their internal distress, which closely resembled the concepts of self-compassion and compassion to others. However, two out the five necessary components of self-compassion were missing, namely understanding the universality of human suffering and tolerance of difficult feelings related to the distress (Strauss et al., 2016); thus, the first-order factors were named self-care and care for others. Both new first-order factors closely reflect strategies already formulated by Litman (2006). For example, self-care could be considered a self-sufficient strategy, and care for others could be considered social support coping. Both strategies are used to lower distress and reduce harm and loss stemming from a stressful event (Carver, 2013a). However, in our data, both self-care and care for others mean more than simply reacting to the suffering. Both coping strategies were also used for balancing the ratio of pleasant and unpleasant emotions experienced by oneself and others so that they feel good about themselves and their lives.

To date, coping strategies of care for others and self-care have been reported multiple times and in numerous settings (e.g., Smalls et al., 2012; Li and Shun, 2016; Li et al., 2019). The individual elements of compassion and self-compassion appear to be helpful in the coping process (e.g., Van Vliet and Kalnins, 2011; Costa and Pinto-Gouveia, 2013; Yu et al., 2016). These topics have mainly been studied in healthcare settings.

### Limitations

Since the sample in Study 2 was non-representative and the participants were selected based on the best coping practices criterium, the results of our qualitative analysis cannot be generalized to the Slovak population. We also focused on understanding how participants with high scores in adaptive coping dealt with distress during the pandemic; it is possible that other coping strategies would emerge if we focused on participants who used non-adaptive rather than adaptive coping. The non-representative sample is also imbalanced in terms of gender, which is a common situation in psychology research (e.g., Davis et al., 2012; Friesen and Williams, 2016).

### Future Research

The low frequency of non-adaptive coping strategies, such as substance use may be related to the fact that participants were selected based on having more adaptive coping strategies. More analysis is needed to find out whether people with a wider variety of both adaptive and non-adaptive coping skills use fewer non-adaptive coping. It would also be beneficial to test the external validity of the English version of The COPE Inventory. Finally, we also suggest revising the COPE Inventory to include the additional two first-order factors that emerged from our qualitative analysis.

## Conclusion

The Slovak version of the COPE Inventory is a reliable, externally valid, and well-structured instrument for measuring coping. In addition to the fifteen first-order factors, it contains three second-order factors: active, social emotional, and avoidance coping.

## Data Availability Statement

In order to comply with the ethics approvals of the study protocols, data cannot be made accessible through a public repository. However, data are available upon request for researchers who consent to adhere to the ethical regulations for confidential data.

## Ethics Statement

The studies involving human participants were reviewed and approved by the Ethical Committee of the Faculty of Social and Economic Sciences at Comenius University in Bratislava. The patients/participants provided written informed consent to participate in this study.

## Author Contributions

MK performed the statistical analysis. All authors designed the research, collected the data, analyzed the data, wrote the manuscript, interpreted the results, revised the manuscript, and read and approved the final manuscript.

## Conflict of Interest

The authors declare that the research was conducted in the absence of any commercial or financial relationships that could be construed as a potential conflict of interest.

## Publisher’s Note

All claims expressed in this article are solely those of the authors and do not necessarily represent those of their affiliated organizations, or those of the publisher, the editors and the reviewers. Any product that may be evaluated in this article, or claim that may be made by its manufacturer, is not guaranteed or endorsed by the publisher.
