# Supplementary material for: The Factor Structure and External Validity of the COPE 60 Inventory in Slovak Translation
Source: Front Psychol. 2022 Feb 28;12:800166. doi: 10.3389/fpsyg.2021.800166 (PMC8918983; doi:10.3389/fpsyg.2021.800166)
Supplement: Supplementary file 1 [file Data_Sheet_1.pdf]

Appendix 1: Factor loadings of fifteen-factor model of the COPE inventory, EFA model with the WLSMV

	COPE01	COPE02	COPE03	COPE04	COPE05	COPE06	COPE07	COPE08	COPE09	COPE10	COPE11	COPE12	COPE13	COPE14	COPE15
C01OPE01	**0.220**	0.009	0.044	-0.011	0.320	-0.011	0.011	0.096	-0.317	0.313	-0.013	-0.010	0.055	0.005	-0.052
C02OPE02	-0.033	**0.210**	-0.016	0.097	0.044	0.060	-0.004	-0.024	-0.062	0.378	0.346	-0.026	0.206	-0.002	0.021
C03OPE03	0.029	0.017	**0.640**	0.140	-0.010	-0.025	-0.041	0.026	0.037	0.249	-0.046	0.058	-0.014	0.022	0.010
C04OPE04	0.087	0.021	-0.071	**0.603**	0.055	-0.166	-0.014	-0.029	0.162	0.291	-0.040	0.017	-0.077	-0.017	0.037
C05OPE05	0.058	-0.127	0.035	0.058	**0.467**	-0.068	0.042	0.054	-0.054	0.338	-0.039	-0.059	0.019	0.103	-0.017
C06OPE06	-0.067	0.041	0.125	0.016	0.008	**0.537**	0.053	0.044	0.024	0.312	0.006	-0.030	-0.060	0.009	0.009
C07OPE07	0.031	-0.019	-0.020	-0.013	-0.038	0.017	**0.972**	0.020	-0.018	0.068	-0.024	-0.027	0.039	0.018	-0.100
C08OPE08	0.026	0.015	0.013	-0.033	-0.002	0.060	0.005	**0.843**	-0.058	0.121	-0.051	-0.027	0.001	0.014	-0.024
C09OPE09	-0.082	0.290	0.173	-0.029	-0.012	0.135	0.031	-0.055	**0.339**	0.238	-0.046	0.110	0.106	-0.102	0.007
C10OPE10	0.093	0.016	-0.070	0.011	0.269	0.029	-0.004	-0.014	-0.014	**0.374**	0.095	-0.004	0.030	0.056	0.039
C11OPE11	0.000	-0.008	0.107	0.785	-0.101	0.011	0.023	0.044	-0.142	0.135	**0.063**	0.019	0.029	-0.001	-0.025
C12OPE12	-0.031	0.045	-0.003	0.012	-0.073	-0.003	0.035	0.018	0.022	0.147	-0.019	**0.905**	0.029	-0.024	-0.017
C13OPE13	-0.008	-0.074	0.010	0.011	0.059	-0.089	0.015	0.024	0.195	0.334	0.034	0.046	**0.547**	0.050	0.001
C04OPE14	-0.021	0.027	-0.040	**0.722**	0.055	0.033	-0.004	-0.018	-0.029	0.242	-0.159	-0.041	0.039	-0.038	-0.066
C14OPE15	0.088	-0.027	0.018	0.003	0.022	0.050	0.002	-0.008	0.024	0.463	0.092	-0.001	0.010	**0.257**	0.236
C02OPE16	0.273	**0.210**	0.076	-0.018	0.046	0.301	-0.043	0.007	0.037	-0.031	0.155	0.062	0.087	-0.103	-0.004
C03OPE17	0.020	-0.013	**0.440**	-0.031	0.389	0.054	-0.022	-0.076	0.291	0.008	0.070	0.061	-0.004	-0.083	-0.137
C07OPE18	0.076	-0.022	0.003	0.039	-0.047	0.025	**0.949**	-0.018	0.028	0.007	-0.010	0.008	0.000	-0.007	-0.043
C15OPE19	0.273	0.119	-0.040	0.018	0.427	0.040	0.004	0.009	-0.069	0.087	0.012	0.030	-0.078	-0.087	**0.012**
C08OPE20	0.067	-0.019	-0.037	0.043	0.058	0.019	-0.030	**0.810**	0.013	-0.069	0.041	0.065	0.022	-0.084	0.004
C13OPE21	0.108	-0.091	0.065	0.015	0.073	-0.009	-0.007	0.045	0.216	0.111	0.098	0.001	**0.479**	0.043	-0.021
C10OPE22	0.366	-0.005	0.038	0.041	0.333	-0.014	-0.027	0.046	0.105	**0.040**	0.075	-0.056	0.033	-0.075	0.075
C11OPE23	0.040	-0.049	0.043	0.694	-0.030	0.133	0.062	0.028	0.058	0.003	**0.219**	-0.038	0.017	-0.028	0.005
C09OPE24	0.058	0.108	0.042	0.058	-0.045	0.109	0.013	0.010	**0.638**	-0.027	0.017	0.066	0.048	-0.023	0.011
C05OPE25	0.240	-0.185	-0.099	0.075	**0.630**	0.052	-0.032	-0.036	0.008	0.076	0.019	0.107	0.045	0.004	-0.045
C12OPE26	0.081	-0.025	0.003	0.022	-0.003	0.058	-0.022	0.016	0.042	-0.063	0.070	**0.874**	0.005	0.021	0.000
C06OPE27	0.033	-0.009	0.079	0.028	0.152	**0.699**	0.046	0.021	0.064	0.032	-0.003	0.030	-0.138	-0.014	0.005
C03OPE28	0.341	-0.015	**0.531**	0.184	0.005	0.091	0.000	-0.025	-0.045	-0.055	-0.017	0.024	0.046	0.058	0.003
C01OPE29	**0.693**	0.008	-0.088	-0.019	0.004	-0.015	0.096	0.040	-0.014	0.027	0.174	-0.053	0.017	0.175	0.044
C04OPE30	0.242	-0.125	0.009	**0.500**	0.228	-0.005	0.003	-0.004	0.147	0.048	-0.039	0.030	-0.076	-0.001	-0.018
C02OPE31	0.084	**0.381**	0.025	0.030	0.093	0.088	-0.008	-0.014	0.026	0.001	0.029	0.160	-0.120	0.163	0.016
C15OPE32	0.010	0.146	0.046	0.013	0.711	-0.106	0.002	0.018	-0.002	0.028	-0.020	-0.019	0.033	0.152	**0.223**
C14OPE33	-0.047	-0.003	0.052	0.018	0.454	0.013	0.024	-0.010	0.152	0.049	-0.079	0.081	-0.061	**0.380**	0.086
C11OPE34	0.012	0.106	0.094	0.591	0.078	-0.036	0.070	-0.028	0.000	-0.053	**0.193**	-0.028	-0.048	0.159	-0.013
C12OPE35	-0.080	0.091	-0.001	0.021	0.064	0.011	0.011	0.033	-0.038	-0.038	-0.003	**0.880**	-0.011	0.033	-0.021
C08OPE36	-0.058	0.025	-0.036	0.033	0.019	0.110	0.002	**0.831**	0.005	-0.016	0.009	0.016	-0.008	0.113	-0.020
C09OPE37	-0.044	0.282	0.018	0.016	-0.025	0.114	0.018	0.030	**0.605**	-0.059	-0.061	-0.025	0.065	0.159	0.010
C01OPE38	**0.473**	0.081	0.024	0.037	0.025	-0.009	0.071	0.016	-0.130	-0.001	-0.036	-0.049	0.077	0.397	-0.022
C15OPE39	0.007	-0.049	0.068	-0.046	0.736	-0.120	0.016	0.064	-0.005	-0.062	0.099	-0.132	0.047	0.163	**0.074**
C06OPE40	0.000	0.258	-0.021	-0.009	-0.017	**0.378**	-0.010	0.137	0.099	0.040	0.023	0.101	-0.055	0.263	0.005
C10OPE41	-0.022	0.041	-0.074	0.024	0.466	0.002	-0.011	-0.009	-0.054	**0.041**	0.063	0.000	0.132	0.273	-0.038
C14OPE42	0.007	-0.018	0.013	0.011	0.324	0.042	-0.015	-0.084	-0.009	0.059	0.080	0.089	0.078	**0.413**	0.082
C02OPE43	0.044	**0.513**	-0.004	0.080	0.005	-0.051	-0.008	0.115	0.022	0.054	0.213	0.049	-0.051	0.050	-0.030
C13OPE44	0.085	0.027	-0.036	-0.014	0.249	-0.074	-0.006	0.016	0.011	0.013	0.021	0.007	**0.497**	0.212	-0.065

C04OPE45	0.006	0.011	-0.072	**0.639**	0.164	-0.028	-0.055	-0.011	0.082	-0.020	-0.070	0.031	0.034	0.177	0.025
C03OPE46	-0.100	0.063	**0.510**	0.114	0.214	0.019	0.049	0.013	0.225	0.015	0.071	-0.081	-0.011	0.019	0.139
C05OPE47	0.020	-0.060	-0.058	0.039	**0.549**	0.049	-0.015	-0.015	-0.167	-0.009	0.024	0.053	0.113	0.030	0.234
C07OPE48	-0.040	0.008	-0.002	0.015	0.040	0.016	**0.913**	-0.038	-0.018	-0.035	0.009	0.035	0.027	0.017	0.097
C10OPE49	0.145	0.077	0.142	-0.005	0.332	-0.072	0.004	-0.011	-0.009	**0.053**	0.001	0.007	0.119	-0.008	0.380
C08OPE50	-0.009	0.005	0.041	-0.031	-0.026	-0.055	0.004	**0.881**	0.011	0.024	0.003	0.029	0.010	-0.041	0.130
C09OPE51	0.050	0.276	0.099	0.023	0.009	0.096	0.093	0.065	**0.212**	0.021	-0.003	0.032	0.014	0.021	0.237
C11OPE52	-0.112	0.007	0.115	0.818	-0.035	-0.001	0.009	0.022	-0.104	-0.037	**0.099**	0.013	0.057	0.015	0.097
C12OPE53	-0.003	-0.007	0.118	-0.075	0.004	-0.014	0.044	0.042	0.008	0.014	-0.034	**0.852**	-0.034	-0.011	0.063
C13OPE54	0.076	0.151	0.018	0.049	0.134	0.006	0.093	-0.007	0.023	0.016	-0.032	-0.059	**0.529**	-0.057	0.057
C14OPE55	-0.022	0.044	-0.015	0.002	0.554	0.061	0.010	0.028	0.188	0.003	-0.153	-0.032	-0.069	**0.125**	0.271
C15OPE56	-0.022	0.001	0.042	0.011	0.777	-0.131	0.032	0.042	0.034	-0.031	-0.005	0.026	0.000	-0.045	**0.122**
C06OPE57	0.080	0.223	-0.077	-0.056	-0.072	**0.383**	-0.007	0.119	-0.002	-0.060	-0.043	0.085	0.148	0.086	0.113
C05OPE58	-0.007	0.042	-0.038	0.014	**0.573**	0.081	0.039	-0.071	-0.176	0.011	0.005	-0.083	0.244	-0.052	0.102
C01OPE59	**0.247**	0.005	0.031	0.052	0.398	0.023	-0.019	0.019	-0.257	-0.025	-0.104	-0.023	0.303	-0.015	0.054
C07OPE60	-0.032	0.032	0.008	-0.009	0.082	-0.044	**0.921**	0.010	0.027	-0.019	0.040	0.037	-0.067	-0.018	0.059
	**0.408**	**0.329**	**0.530**	**0.616**	**0.555**	**0.499**	**0.939**	**0.841**	**0.449**	**0.127**	**0.144**	**0.878**	**0.513**	**0.294**	**0.108**

Notes. COPE01 = Positive reinterpretation and growth. COPE02 = Mental disengagement. COPE03 = Focus on and venting of emotions. COPE04 = Use of instrumental social support. COPE05 = Active coping. COPE06 = Denial. COPE07 = Religious coping. COPE08 = Humor. COPE09 = Behavioral disengagement. COPE10 = Restraint. COPE11 = Use of emotional social support. COPE12 = Substance use. COPE13 = Acceptance. COPE14 = Suppression of competing activities. COPE15 = Planning.

Appendix 2: Factor loadings of fifteen-factor model of the COPE inventory, CFA model with the WLSMV

	COPE01	COPE02	COPE03	COPE04	COPE05	COPE06	COPE07	COPE08	COPE09	COPE10	COPE11	COPE12	COPE13	COPE14	COPE15
C01OPE01	0.752														
C01OPE29	0.689														
C01OPE38	0.741														
C01OPE59	0.790														
C02OPE02		0.691													
C02OPE16		0.543													
C02OPE31		0.422													
C02OPE43		0.447													
C03OPE03			0.749												
C03OPE17			0.723												
C03OPE28			0.750												
C03OPE46			0.712												
C04OPE04				0.824											
C04OPE14				0.822											

C04OPE30	0.783											
C04OPE45	0.741											
C05OPE05		0.757										
C05OPE25		0.803										
C05OPE47		0.702										
C05OPE58		0.749										
C06OPE06			0.736									
C06OPE27			0.749									
C06OPE40			0.701									
C06OPE57			0.595									
C07OPE07				0.937								
C07OPE18				0.967								
C07OPE48				0.941								
C07OPE60				0.918								
C08OPE08					0.841							
C08OPE20					0.856							
C08OPE36					0.853							
C08OPE50					0.875							
C09OPE09						0.790						
C09OPE24						0.762						
C09OPE37						0.721						
C09OPE51						0.650						
C10OPE10							0.642					
C10OPE22							0.690					
C10OPE41							0.677					
C10OPE49							0.659					
C11OPE11								0.851				
C11OPE23								0.818				
C11OPE34								0.848				
C11OPE52								0.856				
C12OPE12									0.920			
C12OPE26									0.921			
C12OPE35									0.937			
C12OPE53									0.918			
C13OPE13										0.754		
C13OPE21										0.678		
C13OPE44										0.749		
C13OPE54										0.729		
C14OPE15											0.645	
C14OPE33											0.619	
C14OPE42											0.678	
C14OPE55											0.636	
C15OPE19												0.640
C15OPE32												0.772
C15OPE39												0.809

C15OPE56	0.771

Notes. COPE01 = Positive reinterpretation and growth. COPE02 = Mental disengagement. COPE03 = Focus on and venting of emotions. COPE04 = Use of instrumental social support. COPE05 = Active coping. COPE06 = Denial. COPE07 = Religious coping. COPE08 = Humor. COPE09 = Behavioral disengagement. COPE10 = Restraint. COPE11 = Use of emotional social support. COPE12 = Substance use. COPE13 = Acceptance. COPE14 = Suppression of competing activities. COPE15 = Planning.

Appendix 3: Examples of suggested dimensions of COPE - the external validation

SELF-CARE

I took a vacation. I got some rest, I sunbathed, and I came back home right before we went into a lockdown… (laugh). But I did not mind, I enjoyed myself and I felt recharged after my vacation. This feeling will last me for a while until I am able to travel again.

ACCEPTANCE

I had trouble accepting tough situations before. I thought tough times could never happen to me. One thing I learned [during pandemic] is that I can accept difficulties as they arise.

ACTIVE COPING

I broke my leg back in the summer. While my leg was broken, a friend of mine took me to go dancing on the beach in the middle of the night. Outside on gravel-covered banks of river Danube, there was music playing, and he took me there in my wheelchair, and we were dancing while we were sitting. It was great, one just needs to be resourceful sometimes...

BEHAVIORAL DISENGAGEMENT

It used to take me months or even years to understand some hurtful and toxic relational dynamics. I feel like I cannot continue, I cannot do anything, I don’t have energy anymore… (to improve my toxic relationships)

CARE FOR OTHERS

I was focused on encouriging others on the Internet. I have a youtube channel and I was creating videos about what I experienced in different cultures... I was hoping to help people recognize things in their lives that they could be grateful for and that they could appreciate.

DENIAL

I experienced all sorts of difficult situations before but this... not this. I had to call an ambulance for my husband and I was thinking: „this is not real“. What was I telling myself? I remember feeling panicked. And I was telling myself that the fact that this was happening again, it could not be real.

SEEKING EMOTIONAL SUPPORT
I have a friend here in my apartment building, we hang out together, sometimes five out of seven evenings. We visit each other and drink tea. I also have close friends from high school that I reach out to and we go for walks....

SEEKING INSTRUMENTAL SUPPORT
Dealing with uncertainty... the situation was always changing. What was helping me was seeking information. I needed to look for relevant information to read. I needed to be informed and know what was happening, but I avoided conspiracy theories. I needed to understand as much as I could about the situation.

HUMOR
I was unable to taste and smell and we were laughing about me not even knowing what I prepared us for dinner.

MENTAL DISENGAGEMENT
I was handling the tough times by watching tv shows... a lot of tv shows and for hours. For many many hours.

PLANNING
I make a lot of plans. I do not rigidly follow the plans but I do make plans. I say that I make plans to change them. But I need to have a plan in the first place and it needs to be a busy one so when I do not follow through on all of the planned activities, the ones that I do are still worthwhile.

POSITIVE REINTERPRETATION
My life turned upside down, but in a different sense. In the back of my mind I was aware that something was happening, that there were things I was not allowed to do as I was complaint with the restrictions, but most of the time I was simply focused on something else. I have to say that the pandemic was very helpful to me. I did not perceive it negatively because it allowed me to spend a full year with my mother. I was busy with work before and I could not spend time with her besides visiting her on weekends. During the pandemic, we were home together every day and we talked a lot to each other.

RELIGION
The pandemic allowed me to understand that many things that happen are outside of my control and that I need to trust God, believe that He exists, and believe that everything happens for a reason. Because if I do not believe, I am miserable.

RESTRAINT
There were almost no cases in the summer and then the cases increased again in the fall. It was very difficult because we were trying to decide whether to enrol our daughter to attend preschool. It was not an easy decision to make and it continues to be difficult for us. Eventually, we decided to keep her at home due to her health status. That’s what we decided and that’s what we did.

SUBSTANCE USE
We have a glass of wine in the evening.

SUPPRESSION OF COMPETING ACTIVITIES

Not to stress and enjoy the moment. Not to try and solve other things. It helps me when I decide not to focus on other things, worry about other things. Instead of thinking about what was and what will be, I focus on what is here and now. And I also do not think about others and their opinions.

VENTING

We have been friends for many years. We attended elementary, middle, and high schools together. I consider her my other sister. I can come to her and I can share with her. And we laugh and cry together. We are on the same wavelength.

Appendix 4: Slovak version of COPE

COPE

Zaujíma nás, ako ľudia reagujú, keď čelia zložitým alebo stresujúcim udalostiam vo svojom živote. Existuje veľa spôsobov, ako sa pokúsiť vyrovnať sa so stresom. Cieľom tohto dotazníka je, aby ste naznačili, čo obvykle robíte a cítite, keď sa u Vás vyskytnú stresujúce udalosti. Je zrejmé, že rôzne udalosti prinesú trochu odlišné reakcie, ale myslite na to, čo zvyčajne robíte, keď ste pod veľkým stresom. Pokúste sa odpovedať na každú položku osobitne vo svojej mysli nezávisle od každej inej položky. Vyberte si odpovede premyslene, aby boli pre VÁS čo najpravdivejšie. Odpovedzte na každú položku. Neexistujú žiadne „správne“ alebo „nesprávne“ odpovede, preto si vyberte čo najpresnejšiu odpoveď pre VÁS a nie to, čo si myslíte, že by „väčšina ľudí“ povedala alebo urobila. Uveďte, čo obvykle robíte, keď VY zažívate stresujúcu udalosť.

	obvykle to nerobím vôbec	zvyčajne to robím trochu	zvyčajne to robím stredne veľa	zvyčajne to robím veľmi veľa
1. Usilujem sa osobnostne rásť na základe vlastných skúseností.	❑	❑	❑	❑
2. Venujem sa práci alebo iným činnostiam, aby som prestal/a myslieť na veci.	❑	❑	❑	❑
3. Som rozrušený/á a nechám svoje emócie prejaviť sa.	❑	❑	❑	❑
4. Pokúšam sa získať od niekoho radu, čo robiť.	❑	❑	❑	❑
5. Usilujem sa s tým niečo urobiť.	❑	❑	❑	❑
6. Hovorím si : „toto nie je skutočné“.	❑	❑	❑	❑
7. Svoju dôveru vkladám v Boha.	❑	❑	❑	❑
8. Smejem sa na situácii.	❑	❑	❑	❑
9. Priznávam si, že sa s tým neviem vyrovnať a prestávam sa snažiť.	❑	❑	❑	❑
10. Ovládam sa, aby som niečo neurobil/a príliš rýchlo.	❑	❑	❑	❑
11. Diskutujem s niekým o svojich pocitoch.	❑	❑	❑	❑
12. Pijem alkohol alebo užívam drogy, aby som sa cítil/a lepšie.	❑	❑	❑	❑

13. Zvykám si na myšlienku, že sa to stalo.	❑	❑	❑	❑
14. Rozprávam sa s niekým, aby som sa o situácii dozvedel/a viac.	❑	❑	❑	❑
15. Usilujem sa, aby ma iné myšlienky alebo činnosti nevyrušovali.	❑	❑	❑	❑
16. Cez deň snívam o iných veciach, než je táto.	❑	❑	❑	❑
17. Som rozrušený/á a skutočne si to uvedomujem.	❑	❑	❑	❑
18. Hľadám pomoc u Boha.	❑	❑	❑	❑
19. Robím si plán činností.	❑	❑	❑	❑
20. Vtipkujem o tom.	❑	❑	❑	❑
21. Uznávam, že sa to stalo a že sa to nedá zmeniť.	❑	❑	❑	❑
22. Riešim to vtedy, keď to situácia umožní.	❑	❑	❑	❑
23. Snažím sa získať citovú oporu priateľov alebo príbuzných.	❑	❑	❑	❑
24. Vzdávam sa už dosiahnutia svojho cieľa.	❑	❑	❑	❑
25. Podnikám ďalšie kroky, ktoré mi pomôžu zbaviť sa problému.	❑	❑	❑	❑
26. Pokúšam sa uvoľniť na chvíľu pitím alkoholu alebo užívaním drog.	❑	❑	❑	❑
27. Odmietam uveriť, že sa to stalo.	❑	❑	❑	❑
28. Dávam priechod svojim pocitom.	❑	❑	❑	❑
29. Snažím sa vidieť to v inom, pozitívnejšom svetle.	❑	❑	❑	❑
30. Rozprávam sa s človekom, ktorý môže urobiť niečo konkrétne s týmto problémom.	❑	❑	❑	❑
31. Spávam dlhšie než zvyčajne.	❑	❑	❑	❑
32. Pokúšam sa vymyslieť si stratégiu, čo robiť.	❑	❑	❑	❑
33. Ak je to nutné, odkladám ostatné veci, aby som sa vysporiadal/a s týmto problémom.	❑	❑	❑	❑
34. Hľadám u niekoho spriaznenosť a porozumenie.	❑	❑	❑	❑
35. Pijem alkohol, alebo užívam drogy, aby som na to menej myslel/a.	❑	❑	❑	❑
36. Zabávam sa na tom.	❑	❑	❑	❑
37. Vzdávam sa pokusu dosiahnuť, čo chcem.	❑	❑	❑	❑
38. Hľadám niečo dobré v tom, čo sa deje.	❑	❑	❑	❑
39. Rozmýšľam nad tým, ako by som mohol/la problém čo najlepšie vyriešiť.	❑	❑	❑	❑
40. Predstieram, že sa to v skutočnosti nestalo.	❑	❑	❑	❑
41. Zabezpečím, aby sa nič nezhoršilo príliš rýchlym konaním.	❑	❑	❑	❑
42. Usilujem sa, aby mi do riešenia tohto problému nezasahovali iné veci.	❑	❑	❑	❑
43. Idem do kina alebo pozerám TV, aby som na to menej myslel/a.	❑	❑	❑	❑
44. Prijímam skutočnosť, že sa to stalo.	❑	❑	❑	❑

45. Pýtam sa ľudí s podobnou skúsenosťou, čo oni vtedy robili.	❑	❑	❑	❑
46. Cítim sa citovo veľmi rozrušený/á a uvedomujem si, že prejavujem veľa týchto svojich pocitov.	❑	❑	❑	❑
47. Podnikám priame kroky, aby som sa vyhol/la problému.	❑	❑	❑	❑
48. Snažím sa nájsť útechu vo svojom náboženstve.	❑	❑	❑	❑
49. Prinútim sa urobiť to, keď na to nastane vhodný čas.	❑	❑	❑	❑
50. Z tejto situácie si robím srandu.	❑	❑	❑	❑
51. Znižujem úsilie, ktoré vynakladám na riešenie tohto problému.	❑	❑	❑	❑
52. Rozprávam sa s niekým o tom, ako sa cítim.	❑	❑	❑	❑
53. Užívam alkohol alebo drogy, aby mi pomohli dostať sa cez to.	❑	❑	❑	❑
54. Učím sa s tým žiť.	❑	❑	❑	❑
55. Odkladám stranou iné činnosti, aby som sa mohol/la na to sústrediť.	❑	❑	❑	❑
56. Veľa myslím na to, aké kroky podniknúť.	❑	❑	❑	❑
57. Správam sa, akoby sa to vôbec nestalo.	❑	❑	❑	❑
58. Krok za krokom robím, čo musí byť urobené.	❑	❑	❑	❑
59. Z každej skúsenosti sa niečo naučím.	❑	❑	❑	❑
60. Modlím sa viac ako zvyčajne.	❑	❑	❑	❑

Appendix 5: Examples of participants statements for suggested new dimensions of COPE – Self-care and Care for others

Self-care	Care for others
"I took a vacation. I got some rest, I sunbathed, and I came back home right before we went into a lockdown… (laugh). But I did not mind, I enjoyed myself and I felt recharged after my vacation. This feeling will last me for a while until I am able to travel again."	*"I was focused on encouriging others on the Internet. I have a youtube channel and I was creating videos about what I experienced in different cultures... I was hoping to help people recognize things in their lives that they could be grateful for and that they could appreciate."*
"…in fact, you let it be, you say what you want to do with it, or sometimes say nothing, you just admit it and realize it's okay, I don't have to be super perfect at all and it's ok."	*"…but now we have been really solving existential problems for some of them."*
"And the truth towards myself helped me a lot. Just really starting to accept and take all these situations as a starting point and identifying that yes, this is me and this is my working material. That's exactly I am my working material."	*"Don't add more burdens to the people. In return, I also tried to support them in some way. So, to make it more balanced."*
"…that I am not alone in this and that I simply encourage myself that I will manage it and remind	*"…that I make tea for myself, so I do to others as well…"*

myself situations that I have handled in the past and somehow I will suddenly get out of it.”	
“And I really needed it [a two-week youth camp] when I had started dealing with those faculty things, because then there were many of them... So, then it was just such a salt for my soul just being two weeks without a phone, without a watch, without anything, just not solving anything. So, it was a very pleasant experience ... I went into it with all my mind and heart. That I was just in the moment that I just consciously decided that I just want to experience this. That I just want to experience in the fullness and richness of all that is possible.”	*“… they were worried about me, and showed interest in myself, how am I doing, or whether I do need anything, so I, because I did not know how to help them at the time, but I wanted to return back at least something, so I googled all sorts of things when they had a problem, as much as possible because I had a lot of time...”*
“Um, I think I'm starting to draw mainly from a source of brief joy and laughter. And so. I realized this is very, very important. And I try to create such an atmosphere both at work and at home. And therefore, there were so much more of joy and merriness recently.”	*“I have helped my sisters with her wedding... Well, I've seen my sister enjoy it all… That was, it was such a great experience.”*
